# Luts-V: A new simplified score for assessing lower urinary tract symptoms in men

**DOI:** 10.1590/S1677-5538.IBJU.2020.0278

**Published:** 2020-12-20

**Authors:** Caroline Santos Silva, Ueslei Menezes de Araujo, Mateus Andrade Alvaia, Kátia Santana Freitas, Taciana Leonel Nunes Tiraboschi, Cristiano Mendes Gomes, José de Bessa

**Affiliations:** 1 Universidade Estadual de Feira de Santana Departamento de Saúde Coletiva Bahia Brasil Departamento de Saúde Coletiva, Universidade Estadual de Feira de Santana, Bahia, Brasil.; 2 Universidade Estadual de Feira de Santana Faculdade de Medicina Bahia Brasil Faculdade de Medicina, Universidade Estadual de Feira de Santana, Bahia, Brasil.; 3 Universidade de São Paulo Faculdade de Medicina Divisão de Urologia São Paulo Brasil Divisão de Urologia, Faculdade de Medicina, Universidade de São Paulo, São Paulo, Brasil.; 4 Universidade Estadual de Feira de Santana Faculdade de Medicina Divisão de Urologia Bahia Brasil Divisão de Urologia, Faculdade de Medicina, Universidade Estadual de Feira de Santana, Bahia, Brasil.

**Keywords:** Me, Validation Study [Publication Type], Prostate

## Abstract

**Objectives:**

Develop and validate a new and simplified score for evaluating the lower urinary tract symptoms in men.

**Materials and methods:**

We modified the existing visual prostate symptom score, including changes in the images, sequence, and new alternatives, resulting in a new visual score (LUTS visual score-LUTS-V). For the validation of the new tool, we used the International Prostatic Symptom Score as the gold-standard and the new LUTS-V to 306 men. The total IPSS score and the total LUTS-V score of each subject were evaluated to determine the agreement between the two instruments. ROC curve was used to evaluate the diagnostic accuracy and best cut-off of LUTS-V. Sensitivity, specificity, and diagnostic odds ratios were used to describe the diagnostic properties.

**Results:**

The mean age of the participants was 59 [52-87] years. There was a significant correlation between LUTS-V and IPSS. (r=0.72 (p <0.0001). The Bland-Altman analyzes demonstrate good agreement between the two questionnaires (bias=5.6%). LUTS-V demonstrated excellent diagnostic accuracy in detecting the most serious cases with an area under the ROC curve of 83% [78-87%] 95% CI. p <0.001). LUTS-V >4 was the best threshold, with a sensitivity of 74% and specificity of 78%.

**Conclusions:**

LUTS-V is a simple, self-administered tool with a significant discriminatory power to identify subjects with moderate to severe LUTS and may represent a useful instrument for the diagnosis and follow-up of men with urinary symptoms.

## INTRODUCTION

Lower urinary tract symptoms (LUTS) comprise a variety of urinary symptoms and are shared among adult men(1, 2). They can be detrimental to the quality of life of affected individuals and are frequently associated with other clinical conditions such as diabetes, neurological disorders, and erectile dysfunction (3, 4). LUTS are common, hurt the quality of life, and generally justify a diagnostic evaluation and treatment, leading to increased costs for the individual and the community (5, 6).

The Assessment of men with LUTS must be focused and take into account all aspects that might be relevant to the differential diagnosis, enabling the clinician to identify symptom severity and associated bother and recognize those who require a more thorough evaluation (7).

Different guidelines recommend using a validated symptom questionnaire in the initial evaluation of men with LUTS (7, 8). Patient-reported outcome assessments are considered useful tools for characterizing symptom burden and health-related quality of life, and they are playing an increasing role in clinical decision-making (9). The International Prostate Symptom Score (IPSS) is the most widely used questionnaire for evaluating men with LUTS (10). An additional question evaluates the impact of LUTS on quality of life (11). It stratifies patients in terms of symptom severity and may be used to monitor disease progression and response to treatment (12).

The use of patient-reported outcome measures may be limited by their extension or the complexity of their questions and response options. Ideally, they should be as short as possible, enabling easy and rapid completion, which may help expand their usage and improve their accuracy (13).

Patients with a low education level have been demonstrated to have difficulty completing the IPSS accurately (14). The problem in understanding the IPSS questions, even for men with a relatively high education level, often leads patients to ask for help when completing the questionnaire. Low literacy increases the risk of unwarranted interference in patient responses (14, 15). The use of simplified, more accessible instruments has been proposed to ease questionnaire completion and minimize interference (16, 17).

The Visual Prostate Symptom Score (VPSS) created by van der Walt et al., comprises pictograms designed to evaluate three of the seven symptoms evaluated in the IPSS: urinary frequency, nocturia, weak stream and also their impact on the quality of life. The VPSS significantly correlates with IPSS and can be completed with no assistance by a higher proportion of men with limited education, indicating it may be more useful than the IPSS for illiterate patients or men with a low education level (16).

Despite its improved applicability (18), the VPSS has some limitations. According to a 2016 study, items that evaluate nocturia and quality of life were deemed unclear by many participants, and the dark pictogram background was also significantly criticized. Suggested improvements included the use of larger images for the pictograms depicting urinary frequency and nocturia and the inclusion of images depicting urinary urgency (19). Although not previously highlighted, we found additional limitations, including the lack of an option for nocturia zero times, typical values for daytime urinary frequency, such as four micturition/day, are scored as increased frequency. The difficulty in interpreting the micturating flow and the use of multiple pictograms for quality of life results in a “ceiling or floor” effect.

We hypothesized that this pictogram would successfully discriminate more severe LUTS in men with a low burden to the respondents. This study aimed to develop and validate a new simplified visual score for the Assessment of men with LUTS.

## MATERIALS AND METHODS

This study was approved by the Research Ethics Committee of the State University of Feira de Santana under protocol no. 64704017.7.0000.0053, position statement 2.052.761, and all participants provided written informed consent.

By modifying the Visual Prostate Symptom Score (VPSS) (16), we developed a new visual score (LUTS-V). From the VPSS settings, we made the following changes in this new version: a) changes in the size of the images, the sequence and pattern of pictograms; b) inclusion of late response (the possibility of the absence of nocturia was added and grouped the daytime frequencies); c) conceptual adjustments and reduction in the number of options from seven to four for the questions about daytime and nocturnal frequency; d) reduction of possibilities for answering a question about the quality of life from 7 to 3 options. The authors reviewed the LUTS-V score to ensure the original content of the VPSS had been maintained and to detect inconsistencies with the new version. The results were submitted to a committee composed of two urologists specialized in voiding dysfunction, one physical therapist and one nurse.

**Figure 1:**
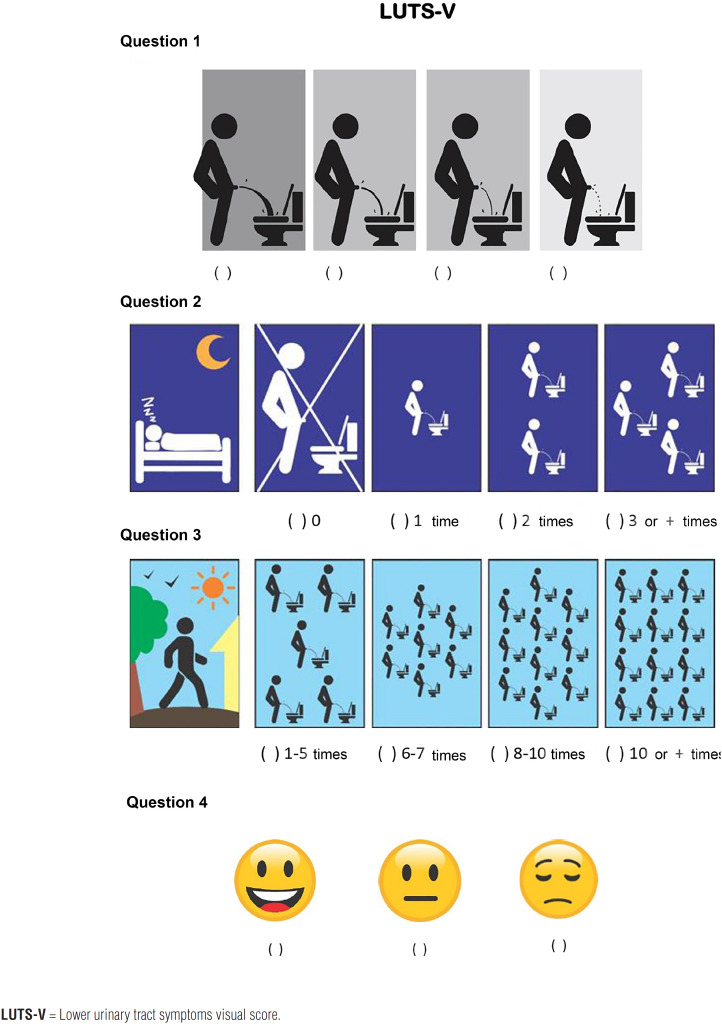
Pictograms in which the patient indicates his assessment of the force of his urinary stream (question 1), urinary frequency during the night (question 2), and day (question 3), and his feelings about his bladder symptoms (quality of life question).

In a pilot study, LUTS-V was administered to 50 men aged >40 years. Respondents were asked about their understanding of the questions and whether the response options were clear. After the committee made minor adjustments, the test version of the instrument was concluded (Figure-1).

Men older than 40 years who presented to a urological outpatient unit met our inclusion criteria. We used the following exclusion criteria: a history of urological surgery in the past 12 months, an acute change in general health status during follow-up. The study cohort consisted of consecutive men who attended urologist office visits between January 2018 and June 2018. Participants were asked to complete both the LUTS-V and the IPSS surveys at baseline.

The questionnaires were self-administered in a private and quiet room. Patients could ask for the assistance of a designated researcher in case of difficulty understanding or completing the surveys. Illiterate men completed the questionnaires in the form of a structured interview. Due to the instrument's visual nature, instructions for participants from different instructional levels did not differ. Additional directions were occasionally necessary for IPSS. After completing LUTS-V, all participants were asked if they had understood each of the items and had found suitable answers.

The COSMIN (Consensus-based Standards for the Selection of Health Status Measurement Instruments) guidelines were used to guide analysis and report (20).

Data were expressed as medians and interquartile ranges, or absolute values and fractions. The Mann-Whitney U test was used to compare continuous variables, while the chi-square and Fisher's exact tests were used to compare categorical variables.

The IPSS (score of 0 to 7 indicates mild symptoms, 8 to 19 indicates moderate symptoms, and 20 to 35 indicates severe symptoms), and LUTS-V (range: 0-11 points) were applied to all subjects.

The total IPSS and LUTS-V scores for each subject were used to determine the agreement between the two instruments using Bland-Altman analysis and Spearman's correlation plot.

A ROC curve was used to evaluate the diagnostic accuracy and the best cut-off point for LUTS-V. Diagnostic properties (criteria validity) were described in terms of sensitivity, specificity, and diagnostic odds ratios.

Uroflowmetry was used as a reference standard for the construct validity analysis of LUTS-V through hypothesis testing and to determine the maximum urinary flow (Qmax). We expected the urinary flow rate to decrease as the total LUTS-V score increased.

ANOVA was used to compare these data and evaluate between-group differences and linear trends.

The time necessary for completion (in minutes) of each questionnaire (IPSS and LUTS-V) was measured to assess the respondent burden.

All tests were two-sided, with a p <0.05 considered statistically significant GraphPad Prism, version 8.03, were used for data analysis.

## RESULTS

**Figure 2:**
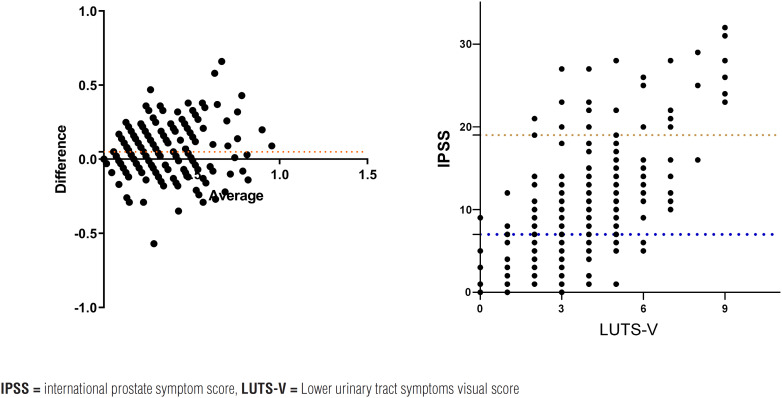
The figure shows the Spearman's correlation between the IPSS and LUTS-V, including quality of life and Bland-Altman plot showing agreement between the IPSS and LUTS-V.

We evaluated 306 men aged 59 [52-67] years, 26 (8.7%) of whom had severe symptoms, while 99 (33%) had moderate symptoms, and 175 (58.3%) had mild symptoms according to the IPSS (Table-1). We found a positive correlation between the IPSS and the LUTS-V total scores (r=0.72; 95% CI: [0.65-0.77]; p <0.0001) (Figure-2), including quality of life (r=0.76; 95% CI: [0.69-0.83]; p <0.0001).

**Table 1:** General and demographic characteristics of 306 patients.

General and demographic characteristics		Median [IQR]
0		59 (52-66)
Schooling in years		11 (8-13.7)
Score do IPSS		6 (3-12)
Score do LUTS-V		3 (2-5)
**Marital status (%)**		
	Not married	11.4%
	Married	80.7%
	Divorced	6.5%
	Widower	1.3%

The Bland-Altman analysis showed good agreement between the two questionnaires (Figure-2) (bias=0.056; p <0.001). Maximum urinary flow rates were found to be significantly lower in moderate and severe cases when compared to those with mild symptoms, i.e., 12mL/s [8-18] and 17 mL/s [13-25], respectively (p <0.001), with a median difference of 5mL/s.

**Figure 3:**
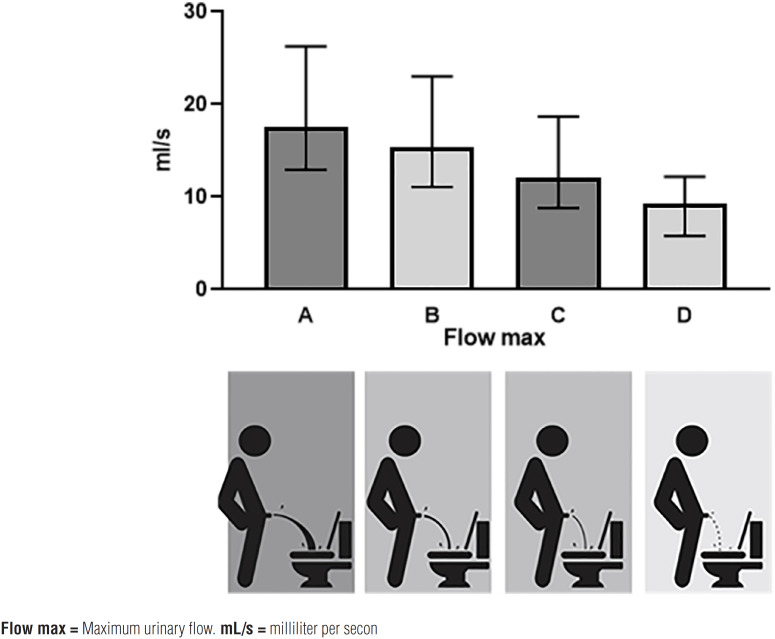
Rates decreased in correlation with the pictograms depicting the urinary stream's force, with the following median maximum urinary flow values.

Furthermore, maximum urinary flow rates decreased in correlation with the pictograms depicting the force of the urinary stream, with the following median Qmax values: A=17.5 [13-16], B=15 [11-23], C=12 [8-18], and D=9.3 [5.7-12.2] mL/s (A to D; p <0.001) (Figure-3).

We found LUTS-V to have excellent diagnostic accuracy in detecting more severe cases, with an area under the ROC curve of 83% (95% CI: [78-87%]; p <0.001). The cut-off value of ≥4 points yielded a sensitivity of 74% and a specificity of 78%. These properties perform a negative predictive value of 81% and a positive predictive value of 71% in this scenario.

Median completion time was 0.51 [0.41-1.07] min for LUTS-V and 2.5 [2.2-3.4] min for the IPSS (p <0.0001). 280 subjects (91.5%) completed both questionnaires without any help, while the other 26 (8.5%) were interviewed. The patients who needed assistance were significantly older (72 [62-74] versus 58 [51-64] years; p <0.001) and had a lower education level (4 [2-7] versus 11 [8-14] years of education; p <0.001).

## DISCUSSION

Simplified questionnaires have been recommended in primary care as screening instruments, particularly for patients with known risk factors, to aid in stratifying a condition and subsequent investigation into the potential worsening of detrimental health issues such as erectile dysfunction (22). The use of simplified instruments to detect LUTS in primary care settings has also been reported (23, 24).

Pictograms have been increasingly used among these simplified instruments. Descazeaud at all. (18), which aimed to validate a French pictogram score for evaluating LUTS, has found comparable diagnostic properties to the IPSS; however, although very similar to the VPSS, the authors included a pictogram for urinary urgency, expanding the scope of symptoms assessed in their score. In a different visual analog scale proposed by Da et al. (25) and termed the GEA scale, the authors used pictograms for all the IPSS questions to test its applicability (completion time and need for assistance).

The high sensitivity and specificity yielded by LUTS-V with a cut-off score of ≥4 points (classified as severe) enable this instrument as a useful screening tool, as it allows for the selective referral of individuals at higher risk to specialized care according to specific guidelines (22).

The completion time for LUTS-V was much shorter than for the IPSS. All men completed the new pictogram, even those who were older and had a lower education level.

We observed an excellent agreement between the quality of life as measured by the IPSS and LUTS-V. Moreover, the reductions of response options improve patient comprehension and reduce the ‘respondent's burden. These results are similar to those found by Crawford and colleagues (26) when developing and validating their simplified instrument UWIN (Urgency, Weak Stream, Incomplete Emptying, and Nocturia), which also contains fewer response options regarding the quality of life.

Uroflowmetry is the most widely used urodynamic study in clinical practice. The maximum urinary flow rate (Qmax) is the most commonly used variable to define voiding dysfunction. Qmax <10mL/s in men has a positive predictive value for detecting obstruction of 88%. Our findings correlate with those of other studies validating the VPSS concerning urodynamic data (27). Similarly to Rogel's research, which validated the Analogical Uroflowmetry tool (ANUF) (28), our urinary stream pictograms were directly correlated with maximum flow rates.

The “LUTS-V” was completed more quickly, who found it easier to understand. Its applicability, use of somewhat entertaining pictograms, and diagnostic properties enable LUTS-V as an alternative to the IPSS and may warrant its full implementation in primary care settings. We believe such actions would considerably benefit men's health, particularly in the screening of more severe cases.

The main limitations of this study were the design of the single-center evaluation, and that contemplate only 3 of the seven questions of the IPSS, including the omission of an item to assess urinary urgency. Therefore, more research is needed to overcome the restrictions mentioned above and to determine the potential advantage of LUTS-V in primary care services with a low prevalence of severe cases and scarce resources.

## CONCLUSIONS

LUTS-V is a simple, self-administered tool with a significant discriminating power to identify patients with moderate to severe symptoms. It may be a useful and quick self-administered alternative tool to the IPSS for the diagnosis and follow-up of men with LUTS.
